# Surface charge change in carbonates during low-salinity imbibition

**DOI:** 10.1038/s41598-024-63317-z

**Published:** 2024-06-06

**Authors:** Felix Feldmann, Emad W. Al-Shalabi, Aksel Hiorth

**Affiliations:** 1 NORCE Norwegian Research Centre, Stavanger, Norway; 2 Research and Innovation Center on CO2 and Hydrogen (RICH), Department of Chemical and Petroleum Engineering, Khalifa University of Science & Technology (KU), Abu Dhabi, UAE; 3 Department of Energy Resources, University of Stavanger, Stavanger, Norway

**Keywords:** Crude oil, Petrol

## Abstract

Optimizing the injection water salinity could present a cost-effective strategy for improving oil recovery. Although the literature generally acknowledges that low-salinity improves oil recovery in laboratory-scale experiments, the physical mechanisms behind it are controversial. While most experimental low-salinity studies focus on brine composition, this study investigated the influence of carbonate rock material on surface charge change, wettability alteration, and spontaneous imbibition behavior. Zeta potential measurements showed that each tested carbonate rock material exhibits characteristic surface charge responses when exposed to Formation-water, Seawater, and Diluted-seawater. Moreover, the surface charge change sensitivity to calcium, magnesium, and sulfate ions varied for the tested carbonate materials. Spontaneous imbibition tests led to high oil recovery and, thus, wettability alteration towards water-wet conditions if the carbonate-imbibing brine system’s surface charge decreased compared to the initial zeta potential of the carbonate Formation-water system. In the numerical part of the presented study, we find that it is essential to account for the location of the shear plane and thus distinguish between the numerically computed surface charge and experimentally determined zeta potential. The resulting model numerically reproduced the experimentally measured calcium, magnesium, and sulfate ion impacts on zeta potential. The spontaneous imbibition tests were history-matched by linking surface charge change to capillary pressure alteration. As the numerical simulation of the laboratory-scale spontaneous imbibition tests is governed by molecular diffusion (with a time scale of weeks), we conclude that molecular diffusion-driven field scale wettability alteration requires several hundred years.

## Introduction

The use of low-salinity injection has been widely studied since the early 1990s. Although the exact mechanisms behind its effects are still not fully understood, it is generally accepted that low-salinity positively impacts oil production in laboratory experiments^1^. Injecting low-salinity brine into carbonates is acknowledged to enhance oil recovery by shifting the wettability towards stronger water-wet conditions^2–6^. Depending on the carbonate core material, Romanuka et al.^7^ proposed two approaches to promote low-salinity effects in carbonates: (A) Modifying the concentration of potential determining ions and (B) Reducing the total ionic strength.

Regardless of changes in the concentration of potential determining ions and/or the reduction of the total ionic strength, several research groups have linked low-salinity effects to changes in the carbonate surface charge. Unlike sandstones, which maintain a fixed surface charge due to the strong covalent bonds in quartz molecules, the crystal structure of carbonates allows for reversible surface charge changes^4^. Several studies indicate that carbonates exhibit a positive surface charge in high-saline brine. However, when modified imbibing brines are introduced into the rock-oil-brine system, the surface charge shifts to negative electrical potentials^8–10^.

Strand et al.^11^ published one of the first studies that investigated the impact of salinity on oil recovery and carbonate surface charge. The authors observed increased spontaneous oil recovery when testing seawater that was enriched with sulfate and calcium ions. Besides increasing spontaneous oil recovery, the admixture of calcium and sulfate ions resulted in stronger negative zeta potential measurements. In a study by Song et al.^5^, zeta potential measurements were conducted for various calcite brine suspensions. The study found that compared to a sodium chloride calcite reference suspension with a zeta potential of approximately + 10 $\text {mV}$, the magnesium enriched and calcium enriched brine calcite suspensions had approximately twice as strong positive electrical potentials. Conversely, suspensions enriched with sulfate ions exhibited strong negative zeta potentials. The findings align with the work of Derkani et al.^10^, who confirmed that calcite particles exhibit a negative zeta potential in deionized water. The increase of calcium and magnesium ion concentration shifted the zeta potential measurements towards stronger positive charges. On the other hand, the increase of sulfate and bicarbonate concentration promoted stronger negative carbonate surface charges.

While several studies emphasized the impact of multivalent ions on carbonate surface charge, other research groups found that introducing highly diluted imbibing brines led to promising low-salinity effects. Yousef et al.^12^ demonstrated the potential of diluted seawater to improve oil recovery in carbonate reservoir cores. After the secondary injection of seawater, the injection of 2, 10, 20, and 100 times diluted seawater resulted in a step-wise oil recovery increase. Compared to two times diluted seawater carbonate system, Yousef et al.^13^ measured a significantly stronger negative zeta potential as the water salinity was further reduced. Jackson et al.^14^ showed that diluted brine limestone systems resulted in less positive zeta potential measurements. Compared to a formation-water and limestone suspension (zeta potential $\approx$ + 6 $\text {mV}$), seawater and limestone (zeta potential $\approx$ − 2 $\text {mV}$) and 20 times diluted seawater and limestone suspension (zeta potential $\approx$ − 8 $\text {mV}$) resulted in stronger negative zeta potential values. Moreover, Jackson et al.^14^ emphasized the importance of the electrical potential between the oil–water interface. Based on streaming potential core floodings, their work concluded that low-salinity effects only occur if the injection water causes a mineral surface charge that is identical (positive or negative) to the electrical potential of the initial oil-water interface.

Although low-salinity effects are understood to occur at the fluid–fluid and rock–fluid interfaces, most reported studies primarily focus on brine composition, crude oil properties, or emulsification^15,16^. The impact of carbonate material on low-salinity mechanism has been widely overlooked. Ferno et al.^17^ investigated the impact of sulfate ions on spontaneous imbibition in Rørdal, Stevns Klint (both deposited during the Maastrichtian age), and Niobrara outcrops chalk samples. The study revealed that higher sulfate concentrations in the imbibing water enhanced oil recovery in Stevns chalk samples. On the other hand, increased sulfate ion concentrations did not affect spontaneous oil recovery in Niobrara and Rørdal chalk samples. Khan et al.^18^ conducted a study to identify suitable outcrop analogs for North Sea formations. While the tested Aalborg, Stevns Klint outcrops, and reservoir samples showed similar porosity, permeability, EDX composition, and pore size distributions, a significant difference in grain texture, surface area, and surface reactivity was found. As a result, the spontaneous imbibition ranged between 0 to almost 80% for the five tested carbonate materials. In a study conducted by Mahani et al.^3^, the researchers investigated the impact of formation-water, seawater, and 25 times diluted seawater on zeta potential measurements of Silurian Dolomite, Island Spar Calcite, Middle East Reservoir Carbonate, and Stevns Klint Chalk. The study results showed that the zeta potential decreased (stronger negative) with decreasing salinity for all four carbonate rock types. However, the surface charge response varied for each material. Compared to Silurian Dolomite and Island Spar Calcite, the Middle East Reservoir Carbonate and especially Stevns Klint Chalk showed significantly stronger negative zeta potential values. The results indicated that each carbonate rock material has a characteristic surface charge response.

In this study, the impact of carbonate rock materials with varying structures, compositions, and diagenesis on surface changes and spontaneous imbibition was investigated by: (1) comparing the sensitivity of different carbonate rock materials to changes in the concentrations of calcium, magnesium, and sulfate ions; and (2) conducting spontaneous imbibition and contact angle measurements to examine if changes in surface charge correlate with the magnitude of wettability changes and spontaneous imbibition behavior. In the numerical part of the presented study, an existing surface complexation model was extended by introducing an exponential distribution to distinguish between the electrical potential at the shear plane and the surface charge. The experimental zeta potential measurements were numerically reproduced, and the spontaneous imbibition tests were history-matched by linking surface charge changes to alterations in wettability and capillary pressure.

## Materials and methods

### Core material

Five carbonate rocks with varying rock mineralogy were selected to investigate the rock material impact on surface charge change and spontaneous oil recovery: Austin Chalk, Edward Limestone, Indiana Limestone, Silurian Dolomite (all outcrops, purchased from Kocurek Industries), and a Reservoir Carbonate. X-ray diffraction analyses were performed on all five rock materials, while BET surface area measurements were conducted on Austin Chalk, Edward Limestone, and Indiana Limestone. The X-ray diffraction analyses and BET surface area results are listed in Table 1, and the core sample properties are summarized in Table 2. While the limestone and chalk samples are pure calcite samples with only minor traces of other minerals, the Austin Chalk and Indiana Limestone material shows a much larger BET surface area than the Edward Limestone. Scanning Electron Microscopy Energy Dispersive X-ray analysis (SEM-EDX) did not indicate clay mineral traces in the five tested carbonate materials.

#### Fluid material

The zeta potential measurements were divided into two measurement sequences. The initial sequence compared the influence of Formation-water, Seawater, and (100-times) Diluted-seawater on carbonate surface potential. The brine compositions of the Formation-water (total dissolved salts (TDS) of 184 $\text {g/L}$), Seawater (TDS of 44 $\text {g/L}$), and Diluted-seawater (TDS of 0.4 $\text {g/L}$) are summarized in Table 3. The second zeta potential test sequence systematically studied the surface charge sensitivity of carbonate rock material on calcium, magnesium, and sulfate ions (brine composition is listed in Table 4).

The spontaneous imbibition tests were performed using a dead light crude oil with a viscosity of 4.2 $\text {cP}$ under ambient conditions. The measured interfacial tension was 27.8 $\text {mN/m}$ for Formation-water and crude oil, 27.3 $\text {mN/m}$ for Seawater and crude oil, and 24.8 $\text {mN/m}$ for Diluted-seawater and crude oil.

### Zeta potential measurement method

The carbonate mineral exposure to brine causes an electrical charge separation at the mineral surface. As a result, the carbonate surface exhibits a definite surface charge that can be experimentally estimated^19^. This study uses the electrophoresis zeta potential measuring principle to evaluate the carbonate surface charge response on brine compositions. During electrophoresis zeta potential tests, the measurement equipment applies an electrical force to stimulate particle movement through the test cell. The particle’s mobility is measured by a laser source that emits light of known amplitude. Once the particles collide with the laser, the emitted light is scattered, which causes an amplitude-frequency shifting. Depending on the magnitude of frequency shifting, the electrophoretic mobility $\text {U}_\text {E}$ is derived. The zeta potential $\zeta$ is then calculated by using Henry’s equation^20^

1 $$\begin{aligned} \zeta = \frac{U_E 3\mu }{2 \varepsilon \varepsilon _{o} f(k \alpha )} , \end{aligned}$$

where $\mu$ is the brine viscosity, $\varepsilon$ is the dielectric constant, $\varepsilon _{o}$ is the vacuum permittivity, and $f(k\alpha )$ relates the particle size to the double layer thickness (here 1.5, Smoluchowski approximation^20^).

Experimental^21^ and numerical^22^ studies have shown that the dielectric constant $\varepsilon$ significantly decreases with increasing ion concentration. Particularly at high ionic strength (Seawater and Formation-water), the decreased dielectric constant impacts the zeta potential calculations. This work used the permittivity model of Mollerup and Breil^22^ to relate the dielectric constant to ionic strength.

Electrophoresis zeta potential measurements require careful and unified sample preparation to obtain reproducible results. After mixing the synthetic brines, the solutions were evacuated for 3 h and sealed from air. On the day of the zeta potential measurements, one gram of dried carbonate powder was mixed with 100 mL brine. The mineral-brine solutions were then sonicated for 5 min at a moderate frequency rate to avoid the overheating of the suspensions. The suspensions were again sealed from air and allowed to equilibrate for approximately five hours.

The zeta potential measurements were conducted with a Malvern Zetasizer Nano ZS, and the tests were run in auto-mode to automatically adapt the applied voltage to the fluid conductivity. The brine-mineral zeta potential results were averaged from eight zeta potential measurement sequences, each comprising 5 to 25 zeta potential sub-measurements.

### Spontaneous imbibition measurement method

The rate of spontaneous imbibition is a measure of a system’s wettability^23^. This study included fifteen spontaneous imbibition tests to compare the impact of carbonate rock material on the primary spontaneous imbibition of Formation-water, Seawater, and Diluted-seawater.

The Indiana Limestone, Edward Limestone, Reservoir Carbonate, Austin Chalk, and Silurian Dolomite samples (diameter of 1.5-inch) were initially CT-scanned and trimmed to 2-inch length. While the outcrop cores were placed inside a soxhlet extractor and exposed to methanol, the reservoir cores were cleaned by an alternating injection of a non-polar (toluene) and polar solvent (methanol). After measuring gas permeability, the samples were vacuum-saturated with connate water and subsequently pressurized for 48 h at 140 $\text {bar}$. Absolute brine permeability was measured at room temperature. The outcrop samples were drained at a centrifuge spin of 12000 revolutions per minute ($\text {RPM}$), and the Reservoir Carbonates were drained at 8000 RPM. Depending on the core material, the connate water saturation (Swc) ranged from 7 (Reservoir Carbonate) to 22.9% (Silurian Dolomite). A static core aging procedure was selected, in which the core samples were aged at 90 $^\circ$C for 30 days. After completing the sample aging, the cores were placed inside Amott cells, surrounded by the selected imbibing brine, and heated to 70 $^\circ$C at ambient pressure. The spontaneous imbibition behavior was monitored for three weeks.

### Contact angle measurement method

Wettability describes the tendency of a fluid to adsorb or detach from a surface under the presence of at least a second immiscible fluid. Contact angle measurements can be used to quantify the wettability of a system^24^. By definition, a contact angle between 0$^\circ$ and 75$^\circ$ is defined as water-wet, a contact angle of 75$^\circ$ to 105$^\circ$ as intermediate-wet, and a contact angle between 105$^\circ$ and 180$^\circ$ as oil-wet^25^. The oil–water–carbonate contact angles were measured using the pendant drop method.

After completing the spontaneous imbibition tests, the oil contact angles of the carbonate–brine–oil systems were measured. The cores were removed from the Amott cells and immersed into a transparent brine-filled container. A crude oil droplet was then placed at the core’s bottom face to measure the system’s contact angle. The contact angles were measured with a Biolin Scientific Optical Tensiometer at ambient conditions.

## Results

### Zeta potential measurements

#### Formation-water, Seawater, and Diluted-seawater zeta potential measurements

**Figure 1:**
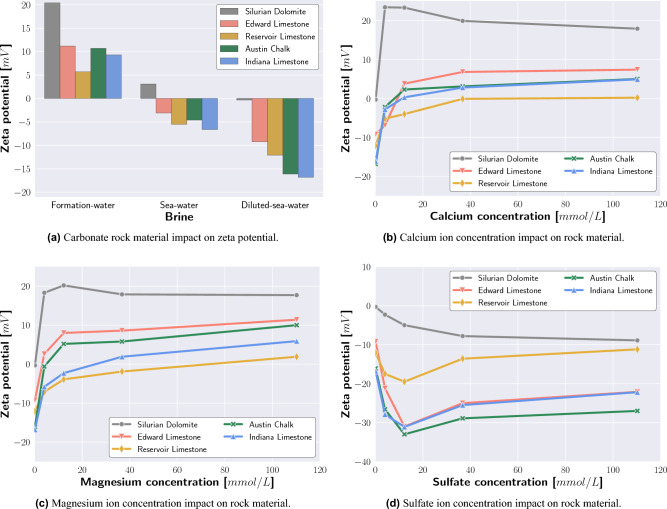
The electrical potential differs for carbonate rock materials when interacting with Formation-water, Seawater, and Diluted-seawater, respectively. Correspondingly, the zeta potential response to calcium, magnesium, and sulfate ion concentration varies for the tested carbonate rock and brine systems.

The electrophoresis zeta potential measurement results of the Formation-water, Seawater, and Diluted-seawater carbonate–brine systems are presented in Fig. 1a and Table 5. In the presence of Formation-water, all tested carbonate–brine systems showed positive electrical potentials. A zeta potential of + 20.4 $\text {mV}$ was measured for Silurian Dolomite and Formation-water, + 11.2 $\text {mV}$ for Edward Limestone and Formation-water, + 10.7 $\text {mV}$ for Austin Chalk and Formation-water, + 9.3 $\text {mV}$ for Indiana Limestone and Formation-water, and + 5.7 $\text {mV}$ for Reservoir Carbonate and Formation-water.

As a result of lower ionic strength and the high sulfate concentration, the Seawater and carbonate systems showed slightly positive (Silurian Dolomite) and negative (Limestone and Chalk) electrical potentials. A zeta potential of + 3.1 $\text {mV}$ was measured for Silurian Dolomite and Seawater, − 3.1 $\text {mV}$ for Edward Limestone and Seawater, − 4.6 $\text {mV}$ for Austin Chalk and Seawater, − 5.5 $\text {mV}$ for Reservoir Carbonate and Seawater, and − 6.6 $\text {mV}$ for Indiana Limestone and Seawater.

Under the absence of divalent ions (Diluted-seawater), all conducted zeta potential measurements resulted in negative values. While Silurian Dolomite and Diluted-seawater caused a zeta potential of − 0.3 $\text {mV}$, strong negative zeta potentials of − 9.2 $\text {mV}$ and − 12.1 $\text {mV}$ were measured for Edward Limestone and Diluted-seawater and Reservoir Carbonate and Diluted-seawater, respectively. The significantly strongest negative zeta potentials of − 16.1 $\text {mV}$ and − 16.8 $\text {mV}$ were measured for Austin Chalk and Diluted-seawater and Indiana Limestone and Diluted-seawater, respectively.

#### Potential determining ion impact on zeta potential

After measuring the Formation-water, Seawater, and Diluted-seawater and carbonate material zeta potential, the subsequent zeta potential test sequence compared the surface charge impact of calcium, magnesium, and sulfate ions on Austin Chalk, Edward Limestone, Indiana Limestone, Silurian Dolomite, and Reservoir Carbonate. As a high saline system interfered with zeta potential measurements, the experiments were constrained to a maximum molar ionic strength of 0.331.

Compared to the calcium-free Diluted-seawater and carbonate systems, Fig. 1b shows that the admixture of small calcium concentration (4 $\text {mmol/L}$) caused an immediate and strong (positive) zeta potential increase. In the case of limestone and chalk, the subsequent calcium concentration increase to 12, 37, and 110 $\text {mmol/L}$, respectively, caused slightly higher positive zeta potentials. Silurian Dolomite exhibited the strongest positive zeta potential at a calcium concentration of 4 $\text {mmol/L}$, while the subsequent calcium concentration increase resulted in a zeta potential reduction.

Similar to calcium ions, a small magnesium concentration (4 $\text {mmol/L}$) caused a significant zeta potential increase, with higher concentrations of magnesium leading to slightly stronger positive electrical potentials (Fig. 1c). Silurian Dolomite exhibited the strongest positive zeta potential at a magnesium concentration of 12 $\text {mmol/L}$, while higher magnesium concentrations resulted in a zeta potential decline.

The impact of sulfate ions on zeta potential is summarized in Fig. 1d. Compared to the sulfate-free reference systems, the admixture of 4 and 12 $\text {mmol/L}$ sulfate ions caused stronger negative zeta potential. A negative zeta potential peak was observed at a sulfate concentration of 12 $\text {mmol/L}$, whereas larger sulfate concentrations (37 and 110 $\text {mmol/L}$) resulted in less negative electric potential measurements. In contrast to the limestone and chalk systems, Silurian Dolomite exhibited a continuous zeta potential decrease as the sulfate ion concentration increased.

In agreement with the Formation-water, Seawater, and Diluted-seawater zeta potential measurements (Fig. 1a), the potential determining ions and carbonate zeta potential measurements emphasized that each carbonate rock has a characteristic surface charge response (Fig. 1b–d). While the overall trend of limestone and brine zeta potential response is consistent, the quantitative zeta potential results varied for the tested rock material.

### Spontaneous imbibition tests

In the first spontaneous imbibition test sequence, the spontaneous imbibition tendency of Formation-water into the five carbonate rocks was measured. A spontaneous oil recovery of 2.4% was observed for Reservoir Carbonate and Formation-water, 1.9% for Indiana Limestone and Formation-water, 0.9% for Edward Limestone and Formation-water, and 0.9% spontaneous oil recovery for Austin Chalk and Formation-water. No spontaneous oil recovery was measured for Silurian Dolomite exposed to Formation-water (Table 2).

**Figure 2:**
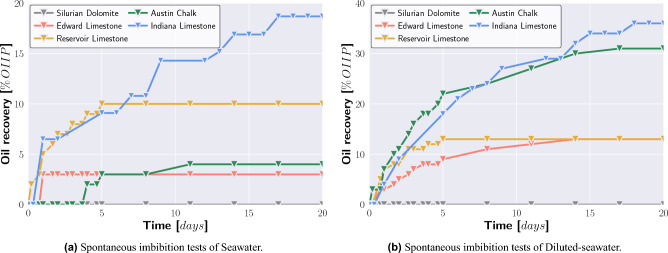
Spontaneous imbibition tests at 70 $^{\circ }$C. Seawater caused moderate spontaneous oil recovery in the case of Indiana Limestone and Reservoir Carbonate. Diluted-seawater caused significant spontaneous oil recovery in the case of Indiana Limestone and Austin Chalk and moderate spontaneous imbibition in the case of Reservoir Carbonate and Edward Limestone.

Figure 2a displays the spontaneous imbibition results of the medium saline and sulfate-rich Seawater. The synthetic Seawater caused a spontaneous oil recovery of 18.7% in the case of Indiana Limestone, 10.1% spontaneous oil recovery in the case of Reservoir Carbonate, 5.2% spontaneous oil recovery in the case of Austin Chalk, and 2.5% spontaneous oil recovery in the case of Edward Limestone. No spontaneous oil recovery was observed when Silurian Dolomite was exposed to Seawater.

The spontaneous imbibition tests of Diluted-seawater are summarized in Fig. 2b. The highest spontaneous oil recovery of 36.1% and 31.3% was measured for Indiana Limestone and Austin Chalk, respectively, while Diluted-seawater caused a spontaneous oil recovery of 13.2% and 12.8% in the case of Reservoir Carbonate and Edward Limestone. No spontaneous oil recovery was observed when Silurian Dolomite was exposed to Diluted-seawater.

### Contact angle measurements

**Figure 3:**
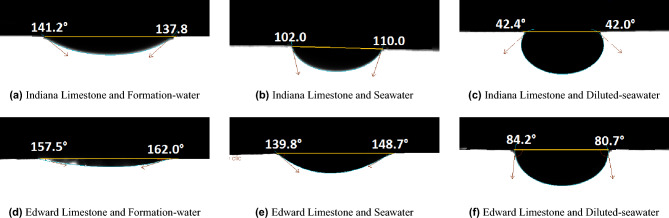
Oil contact angle measurements of Indiana Limestone and Edward Limestone using Formation-water, Seawater, and Diluted-seawater, respectively, after spontaneous imbibition. The measured oil contact angle decreased with decreasing salinity.

The oil contact angle results for Indiana Limestone and Edward Limestone using Formation-water, Seawater, and Diluted-seawater are displayed in Fig. 3. When Formation-water was used, all five carbonate materials showed strong oil-wet contact angles in a range of 170.5 to 156.2$^\circ$ (Table 2, Fig. 3a,d).

Compared to Formation-water, the carbonate exposure to Seawater caused less oil-wet conditions. An oil contact angle of 118.4$^\circ$ was measured for Indiana Limestone and Seawater (Fig. 3b), 121.0$^\circ$ for Austin Chalk and Seawater, 134.6$^\circ$ for Edward Limestone and Seawater (Fig. 3e), and 143.7$^\circ$ for Reservoir Carbonate and Seawater. The measured contact angle of Silurian Dolomite and Seawater was 161.5$^\circ$.

The spontaneous imbibition tests resulted in the significantly highest oil recovery when Diluted-seawater was used as imbibing water. In the presence of Diluted-seawater, a contact angle of 59.2$^\circ$ was measured for Indiana Limestone (Fig. 3c), 91.6$^\circ$ for Edward Limestone (Fig. 3f), 119.3$^\circ$ for Austin Chalk, and 124.2$^\circ$ for Reservoir Carbonate. Moreover, a contact angle of 155.0$^\circ$ was observed for Silurian Dolomite and Diluted-seawater. The contact angle measurements showed a wettability alteration towards a more water-wetting state as the water salinity decreased in agreement with the zeta potential and spontaneous imbibition tests.

### Surface complexation modeling

The exposure of carbonate to polar fluids leads to chemical reactions at the mineral’s surface, e.g., *ionization*, *dissociation*, *ion dissolution*, and *ion adsorption*^26^. While the irreversible *ionization* predominantly leads to the generation of new chemical compounds, the reversible *dissociation* causes the breaking of molecules into smaller components. At the calcite surface, *dissociation* separates the crystal calcium carbonate structure. The magnitude of *ionization* and *dissociation* is significantly influenced by the pH value of the suspension. Moreover, the presence of aqueous calcium and carbonate ions can cause an *ion dissolution* of the crystal calcite structure. In addition to *ion dissolution*, the brine composition affects the *ion adsorption* from the electrolyte solution. Depending on the adsorption preference and ion concentration, the attraction of cations (e.g., calcium, magnesium) or anions (e.g., sulfate, carbonate) promotes positive or negative surface charges^26^.

**Figure 4:**
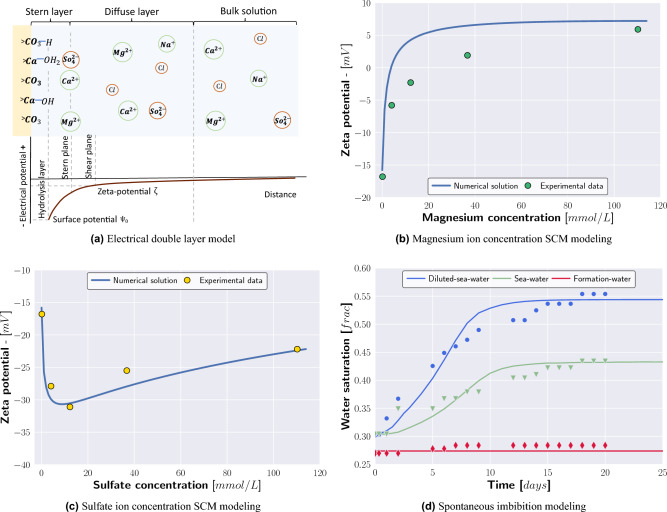
Electric double layer layer model for calcite surfaces (**a**). The surface complexation model captures the trend of zeta potential measurement for the magnesium and sulfate enriched brines, respectively, and Indiana Limestone systems (**b,c**). The numerical model reproduces the spontaneous imbibition of the Diluted-seawater, Seawater, and Formation-water and Indiana Limestone tests (**d**).

The combined chemical processes result in a surface charge that is compensated by the generation of two electrical layers around the mineral surface, typically described by an Electrical double layer model (EDL)^27–30^. Due to the exposure of the calcite mineral surface to brine, the functional groups of the calcite lattice ($\text {Ca}^\text {2+}$) and ($\text {CO}_\text {3}^\text {2-}$) interact with the brine ions. Near the calcite surface, the functional calcite groups chemically bond hydron ($\text {H}^+$) and hydroxide ($\text {OH}^-$) (Fig. 4a). However, the majority of surface charge compensation occurs at the Stern plane, where potential determining ions are attracted^31–33^. Besides attracting multivalent ions, the Stern plane separates the Stern layer from the Diffuse layer, in which Coulomb forces cause a loose attraction of co- and counter-ions. In contrast to the Stern plane, where only multivalent ions attach, the diffuse layer contains monovalent ions such as sodium, potassium, and chloride^33,34^.

Formation-water and Seawater are characterized by significant divalent ion accumulation at the Stern layer. As a result of the potential-determining ion attraction, the calcite surface charge compensation occurs over a short distance. Handy et al.^35^ suggested that a Seawater calcite Electrical Double Layer (EDL) length falls within the nanometer range compared to the micrometer range for deionized water.

To numerically reproduce the zeta potential measurements described in the “Zeta potential measurements” section, a surface complexation model previously developed for calcite surfaces was used and extended^36,37^. The model is integrated into the NORCE Norwegian Research Centre in-house core scale simulator IORCoreSim, as well as the field scale simulator IORSim (a collaborative development of Institute for Energy Technology, University of Stavanger, and NORCE). The used surface complexation model accounts for the following surface reactions:

2 $$\begin{aligned} {>\text {CaCO}_{3}^{-}}&\rightleftharpoons >\text {CaH}_{2}\text {O}^{+}+ {\text {HCO}_3^-}- {\text {H}^{+}}-{\text {H}_2\text {O}}, \end{aligned}$$

3 $$\begin{aligned} {>\text {CaOH}^{0}}&\rightleftharpoons >\text {CaH}_{2}\text {O}^{+}- {\text {H}^{+}}, \end{aligned}$$

4 $$\begin{aligned} {>\text {CaHCO}_3^{0}}&\rightleftharpoons >\text {CaH}_{2}\text {O}^{+}+ {\text {HCO}_3^-}- {\text {H}_2\text {O}}, \end{aligned}$$

5 $$\begin{aligned} {>\text {CaSO}_4^{-}}&\rightleftharpoons >\text {CaH}_{2}\text {O}^{+}+ {\text {SO}_{4}^{2-}}, \end{aligned}$$

6 $$\begin{aligned} {>\text {CO}_{3}\text {H}^{0}}&\rightleftharpoons {>\text {CO}_3^{-}}+ {\text {H}^{+}}, \end{aligned}$$

7 $$\begin{aligned} {>\text {CO}_3\text {Ca}^{+}}&\rightleftharpoons {>\text {CO}_3^{-}}+ {\text {Ca}^{2+}}, \end{aligned}$$

8 $$\begin{aligned} {>\text {CO}_3\text {Mg}^{+}}&\rightleftharpoons {>\text {CO}_3^{-}}+ {\text {Mg}^{2+}}, \end{aligned}$$

where the surface charge density $\sigma$ is determined by the sum of the positive and negative surface complexes^38^, given by

9 $$\begin{aligned} \frac{S_A}{\mathscr {F}}\sigma = [>\text {CaH}_{2}\text {O}^{+}]+[{>\text {CO}_3\text {Ca}^{+}}] +[{>\text {CO}_3\text {Mg}^{+}}] -[{>\text {CaCO}_{3}^{-}}] -[{>\text {CO}_3^{-}}] -[{>\text {CaSO}_4^{-}}], \end{aligned}$$

where $\text {S}_\text {A}$ is the specific surface area, $\mathscr {F}$ is the Faraday constant, and the brackets denote the surface complexion concentrations. The numerical surface charge density can be adjusted by tuning the reactions constant of Reaction (2) to (8), in the following example described for the law of mass action of Reaction (8)^38^:

10 $$\begin{aligned} log_{10} K= log_{10} [{>\text {CO}_3^{-}}] + log_{10} \,\alpha _{Mg^{2+}} - log_{10} [{>\text {CO}_3\text {Mg}^{+}}] +2\frac{\mathscr {F}\psi _o}{ln10 RT}, \end{aligned}$$

where $\alpha$ denotes the thermodynamic activity, $\psi _\text {o}$ is the surface potential, $\text {R}$ is the ideal gas constant, and $\text {T}$ is temperature. The relationship between the surface potential $\psi _\text {o}$ and the surface charge density $\sigma$ is given by the Grahame equation as follows

11 $$\begin{aligned} \sigma ^2 = 2000\varepsilon \,\varepsilon _o\,R\,T \displaystyle \sum _{i} n_i^B \left[ \exp {\left( \frac{-z_i\mathscr {F}\psi _0}{RT}\right) }-1\right] , \end{aligned}$$

where $\text {n}_\text {i}^\text {B}$ is the molar concentration of charged species number $\text {i}$ in the bulk aqueous phase and $\text {z}_\text {i}$ denotes the charge number of ion $\text {i}$. While a comprehensive description of solving the surface complexation model is provided in Nødland and Hiorth^38^, the computed surface potential does not necessarily represent the experimentally measured zeta potential measurements. To improve the comparability of the zeta potential measurements and the numerical solved surface potential, an exponential distribution is introduced to approximate zeta potential from surface potential^39–42^

12 $$\begin{aligned} \zeta = \psi _0 \exp \left( \frac{-\lambda _\zeta }{\lambda _{D}}\right) , \end{aligned}$$

where $\zeta$ is the zeta potential, $\lambda _\zeta$ is the distance to the Shear plane and $\lambda _\text {D}$ denotes the Debye length. Although the Shear plane distance is a function of the solution’s salinity, a constant value of $1^{-10}$ m was assumed, which is in agreement with the Shear plane distance suggested in Refs.^41,42^. The Debye length is calculated as follows^43^

13 $$\begin{aligned} \lambda _{d}=\left( {\frac{\varepsilon \varepsilon _o k_B T}{ 2e^2N_A I}}\right) ^{1/2}, \end{aligned}$$

where $\text {k}_\text {B}$ is the Boltzmann constant, $\text {e}$ is elementary charge, $\text {N}_\text {A}$ is the Avogadro constant, and $\text {I}$ is the ionic strength, which is defined as

14 $$\begin{aligned} I=\frac{1}{2}\sum _{i=1}^{n}c_{i}z_{i}^{2}, \end{aligned}$$

where $\text {c}_\text {i}$ is the molar concentration of ion i and $\text {z}_\text {i}$ denotes the charge number of ion $\text {i}$. The dielectric constant $\varepsilon$ decreases with increasing salinity, which was captured using a model developed for electrolyte solutions^22^.

Figure 4b,c show the measured and modeled impact of magnesium and sulfate ions on zeta potential measurements for the Indiana Limestone samples. The surface complexation modeling supports that with increasing magnesium concentration, the initially negative zeta potential increases with increasing magnesium ion concentration. While small sulfate concentration caused a significant zeta potential decrease, the SCM model replicates the trend of a slightly stronger positive zeta potential as the sulfate concentration (and hence overall salinity) exceeds a threshold concentration (Fig. 4c). The used dimensionless equilibrium constants (reference temperature of 25 $^{\circ }$C) are listed in Table 6.

After history matching the calcium, magnesium, and sulfate zeta potential curves, the surface complexation model estimated a zeta potential of 5.9 $\text {mV}$, − 5.5 $\text {mV}$, − 15.7 $\text {mV}$ for Indiana Limestone and Formation-water, Seawater, and Diluted-seawater, respectively, which is in good agreement with the experimentally determined zeta potentials (Table 5). To address the varying response of limestone material to brine composition as depicted in Fig. 1, the SCM model could, for instance, permit adjustments to the number of functional calcite groups $\text {Ca}^\text {2+}$ and $\text {CO}_\text {3}^{2-}$, or allow minor tuning of the surface equilibrium constants.

**Figure 5:**
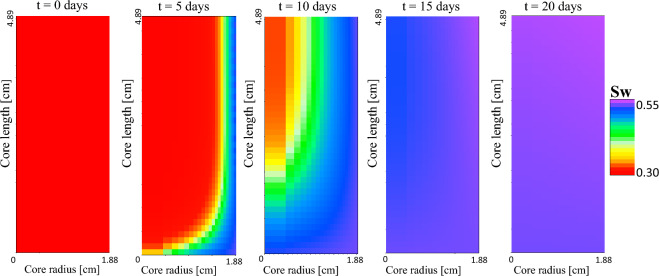
Radial water saturation development of sample IL3 displayed from the center of the core towards the outer radial face. The Diluted-seawater imbibes through the outer radial face, slowly replacing the mobile oil that migrates towards the core’s top front (from left to right).

### Spontaneous imbibition modeling

After numerically reproducing the impact of potential determining ions on surface charge, the spontaneous imbibition tests of Indiana Limestone and Formation-water, Seawater, and Diluted-seawater as imbibing brine were history-matched. The numerical spontaneous imbibition model used Dirichlet boundary conditions along the radial outer and top faces, maintaining a fixed atmospheric pressure and constant water saturation of 1. As the core samples were directly placed on the bottom of the Amott cell, the bottom core face was represented by a no-flow boundary. Moreover, the core properties were assumed to be homogeneous, and the water imbibition process was driven by interpolating between an oil-wet and a water-wet capillary pressure curve given by Sandvik^44^

15 $$\begin{aligned} Pc_{in}= F \cdot Pc_{ww}(S_w) + (1-F) \cdot Pc_{ow}(S_w), \end{aligned}$$

where $\text {Pc}_\text {in}$ is the interpolated capillary pressure, $\text {F}$ is the normalized salinity with $\text {F} = \text {x} \in \mathbb {R} [0,1]$, $\text {Pc}_\text {ow}$ denotes the oil-wet capillary pressure, and $\text {Pc}_\text {ww}$ denotes the water-wet capillary pressure. For a high saline (and hence positive zeta potential) system, the interpolation parameter F approached 0. Conversely, a low saline (and thus negative zeta potential) system was reflected by an interpolation parameter F tending towards 1. Based on the comprehensive Indiana Limestone capillary pressure measurement data set provided in Refs.^45,46^, a three-constant hyperbolic function was selected to extrapolate water-wet and oil-wet capillary pressure curves to the Indiana Limestone samples as follows^47^

16 $$\begin{aligned} Pc= \frac{B + C S_w}{1+D S_w}, \end{aligned}$$

where the parameters *B*, *C*, and *D* were modified during the numerical history matching to replicate the spontaneous imbibition of Seawater and Diluted-seawater, respectively, into the Indiana Limestone samples. The shift in zeta potential and, hence, in capillary pressure is governed by molecular diffusion of ions into and out of the cores. The diffusion of molecules within the liquid phases follows Fick’s law, which is described as follows^44^:

17 $$\begin{aligned} J^k_w= -\nabla c_w^k \rho _w D_w^k \phi ^m S_w^n, \end{aligned}$$

where $\text {J}^\text {k}_\text {w}$ is the diffusive flux of component $\text {k}$ in the water phase, $\text {c}_\text {w}^\text {k}$ is the fractional concentration of component $\text {k}$, $\rho _\text {w}$ is the water density, $^\text {m}$ is the cementation exponent, and $^\text {n}$ is the saturation index. Using Fick’s law of diffusion, it is furthermore possible to estimate the time required for molecular diffusion across a specified spatial interval. The spontaneous imbibition models assumed a salt bulk diffusion coefficient of $3\times 10^{-9}$ $\text {m}^2/\text {s}$, which corresponds to a molecular diffusion distance of approximately 1 $\text {cm}$ within 25 days (with $^\text {m}$ = 2.3, $^\text {n}$ =1.9)

18 $$\begin{aligned} t=\frac{L^2}{4 \cdot D_w^k \cdot \rho _w D_w^k \phi ^m S_w^n}. \end{aligned}$$

The spontaneous imbibition modeling results are plotted in Fig. 4d and show a good replication of the Seawater and Diluted-seawater and Indiana Limestone SI tests. The magnitude of the spontaneous imbibition process is controlled by two input parameters: the shape of the capillary pressure curves and the effective diffusion coefficient of the salt dissolved inside the brine. Using a constant bulk diffusion coefficient, the capillary curves remain to fit and history-match the spontaneous oil recovery.

**Figure 6:**
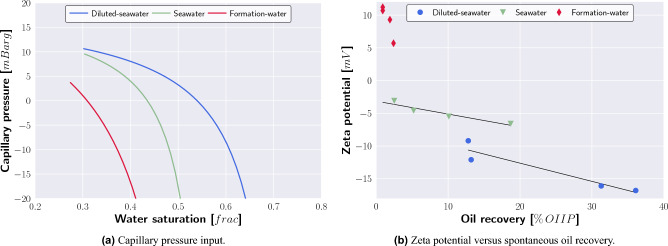
Compared to Formation-water as imbibing fluid, the capillary pressure Seawater and Diluted-seawater curves are characterized by stronger water-wet conditions (**a**). The comparison of the spontaneous imbibition and zeta potential measurements demonstrates that high spontaneous oil recovery was obtained if the brine-carbonate systems were characterized by strong negative zeta potentials (**b**).

The water saturation development of the sample IL3 (Diluted-seawater as imbibing fluid (Table 2)) is displayed in Fig. 5. The water imbibes through the outer radial face, slowly replacing the mobile oil that migrates towards the core’s top face. The modeled water saturation development agrees with the experimental observation, where oil droplets were predominantly observed on the core top face. Note that, Fig. 5 plots the cross-section from the core center radially towards the outer core face. The corresponding capillary pressure curves of Diluted-seawater and Seawater simulations, respectively, are plotted in Fig. 6a.

## Discussion

In the first zeta potential test series, the impact of Formation-water, Seawater, and Diluted-seawater on the Surface charge of carbonates was investigated. Positive zeta potentials were measured for Formation-water and carbonates, slightly negative zeta potentials for Seawater and limestone/chalk, and strong negative zeta potentials were measured for Diluted-seawater and carbonate. Moreover, calcium and magnesium ions were demonstrated to promote positive electrical potential, whereas sulfate ions induce stronger negative calcite surface charges. While the tested limestone materials showed a consistent trend, the quantitative zeta potential results and surface charge sensitivity varied across the tested core materials.

The Surface complexation model developed by Van Cappellen et al.^36^ and Hiorth et al.^37^ was extended by approximating zeta potential from surface potential using an exponential distribution. After numerically validating the measured surface complexation response of calcium, magnesium, and sulfate ion concentration on the different carbonate materials, the experimental zeta potential results of Formation-water, Seawater, and Diluted-seawater and carbonates systems were numerically reproduced.

Although the X-ray diffraction and SEM-EDX characterized the Austin Chalk, Edward Limestone, Reservoir Carbonate, and Indiana Limestone material as pure calcite material, the zeta potential measurements showed that each mineral has a characteristic surface charge sensitivity. The presented SCM model captured the characteristic surface charge sensitivity by modifying the number of functional calcite groups $\text {Ca}^\text {2+}$ and $\text {CO}_\text {3}^{2-}$.

Based on BET surface area measurements, the mineral surface area of the tested samples ranged from 30 to 90 $\text {m}^2$, while the saturated cores contained between 8 and 18 $\text {ml}$ of fluid. On average, 1 $\text {mL}$ of fluid is surrounded by 5 $\text {m}^2$ of rock surface. Considering the substantial differences between rock surface and fluid volume proportions, it is essential to emphasize the significant influence of core material on spontaneous imbibition rates. Figure 6b shows that high spontaneous oil recovery was obtained for brine-carbonate systems characterized by strong negative zeta potentials. In accordance with the spontaneous imbibition tests, the initially highly oil-wet contact angles decreased as Seawater and Diluted-seawater were used as imbibing fluid (Fig. 3).

**Figure 7:**
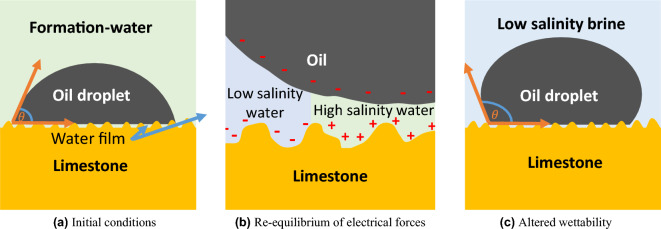
Wettability alteration/spontaneous imbibition sketch based on surface charge in carbonates. The wettability alteration process assumes the low-salinity water diffusion into the water film between the carbonate surface and oil phase (**a**). As a result of surface charge change, the repulsive forces between the oil and carbonate surface are strengthened (**b**). The wettability is changed towards stronger water-wet conditions (**c**) (modified after^4^).

When simulating the implemented spontaneous imbibition tests with a Darcy-scale (continuum-scale) simulator, it is challenging to correlate the impact of surface complexation modeling with changes in fluid flow. To gain a better understanding of pore-scale processes, we recommend using a Lattice Boltzmann solver coupled with a geochemical solver, for instance, the open-source BADChIMP software that was used in Hiorth et al.^48^ and is available in Ref.^49^. In this study, we postulate that wettability, and consequently capillary pressure, changes in response to changes in the magnitude of carbonate surface charge. Positive surface potential was attributed to an oil-wet capillary pressure curve. When the brine-mineral system was exposed to Seawater or Diluted-seawater, the surface charge shifted towards a negative potential, resulting in an interpolation of oil-wet and water-wet capillary pressure curves.

The assumption is based on a mechanism proposed by Mahani et al.^4^. Under the presence of high saline Formation-water and positively charged carbonate surface, acid oil tends to coat the carbonate surface (Fig. 7a). During the spontaneous imbibition tests with Seawater and Diluted-seawater, the less saline imbibing water diffuses into the water film between the oil phase and the carbonate surface. The diffusion leads to a decrease in calcium and magnesium ion concentration, prompting a re-equilibration of the carbonate surface charge towards a less positive electrical potential. The reduction in (positive) surface charge can be magnified by the presence of sulfate anions, as demonstrated by the zeta potential measurements. Figure 7b,c sketch the re-equilibrium of the oil-brine-rock system, in which the negative surface charge generates repulsive forces between the carbonate surface and acid oil components. The stability of the water film along the carbonate pore surface can be further enhanced by the reduction in total ionic strength, leading to an expansion of the electrical double layer (EDL)^4^. The EDL length in the presence of seawater falls within the nanometer range, compared to the micrometer range observed with deionized water^35^. As demonstrated in the study, the magnitude of wettability alteration and spontaneous imbibition behavior varies with mineralogy and depends on the carbonate’s surface charge sensitivity towards salinity and divalent ions.

Despite over three decades of research proposing various mechanisms for low-salinity effects and numerous documented successes in improving spontaneous imbibition at the laboratory scale, the number of field-scale applications remains limited. In the numerical spontaneous imbibition model, the rate of wettability change (capillary pressure) is controlled by molecular ion diffusion. The utilized salt bulk diffusion coefficient of $3\times 10^{-9}$ $\text {m}^2/\text {s}$ corresponds to an effective molecular diffusion distance of 1 $\text {cm}$ per 25 $\text {days}$, or 1 $\text {m}$ per 7 $\text {years}$. Consequently, diffusion-driven wettability alteration at the field scale requires several hundred years. Although laboratory spontaneous imbibition tests may demonstrate promising recovery potential, the corresponding diffusion-driven wettability changes at the field scale are economically unattractive. To assess the field potential of low-salinity flooding, we recommend implementing core flooding tests, where wettability alteration is investigated alongside viscous forces.

**Table 1:** X-ray diffraction analysis of Austin Chalk, Edward Limestone, Indiana limestone, Reservoir Carbonate, and Silurian Dolomite.

Sample	Calcite [%]	Dolomite [%]	Quartz [%]	BET surface area [$\text {m}^2/\text {g}$]
Austin Chalk	99.6	0	0.4	0.835
Edward Limestone	99.6	0	0.4	0.274
Indiana Limestone	98.5	0.7	0.8	0.541
Reservoir Carbonate	98.9	0.9	0.2	
Silurian Dolomite	0	99.2	0.8	

Brunauer–Emmett–Teller (BET) surface area measurements of Austin Chalk, Edward Limestone, Indiana Limestone.

**Table 2:** Core properties, spontaneous imbibition, and contact angle measurement results of the Indiana Limestone (IL), Edward Limestone (EL), Reservoir Carbonate (RC), Austin Chalk (AC), and Silurian Dolomite (SD) samples.

	IL1	IL2	IL3	EL1	EL2	EL3	RC1	RC2	RC3	AC1	AC2	AC3	SD1	SD2	SD3
Brine perm [mD]	8.6	6.2	8.5	12.4	12.5	12.3	35.7	10.3	8.6	14.0	11.5	17.9	3.0	44.2	24.1
Porosity [%]	15.0	14.9	15.6	23.5	23.7	23.3	31.4	29.9	30.9	24.8	25.5	26.2	12.7	13.8	13.7
Swc [%]	27.0	30.4	30.2	12.1	9.8	10.0	13.2	15.5	7.0	17.0	17.6	19.7	18.4	19.8	22.9
Imbibing water	FW	SW	DSW	FW	SW	DSW	FW	SW	DSW	FW	SW	DSW	FW	SW	DSW
SI recovery [%]	1.9	18.7	36.1	0.9	2.5	12.8	2.4	10.1	13.2	0.9	5.2	31.3	0	0	0
Contact angle [$^\circ$]	156	118	59	161	135	92	165	144	124	170	121	119	171	162	155

The imbibing fluids are Formation-water (FW), Seawater (SW), and Diluted-seawater (DSW).

**Table 3:** Brine composition in mmol/L of Formation-water, Seawater, and Diluted-seawater brine.

Brine	$\text {Na}^+$	$\text {K}^+$	$\text {Ca}^{2+}$	$\text {Mg}^{2+}$	$\text {Sr}^{2+}$	$\text {Cl}^-$	$\text {Br}^-$	$\text {SO}_\text {4}^{2-}$	TDS [$\text {g/L}$]	Molar ionic strength
Formation-water	2237	23	372	84	9	3165	9	4	183.4	3.66
Seawater	587	13	13	65	–	683	–	37	43.9	0.87
Diluted-seawater	5.9	0.13	0.13	0.65	–	6.8	–	0.37	0.4	0.0087

**Table 4:** Brine composition in mmol/L and corresponding molar ionic strength of calcium, magnesium, and sulfate, respectively, enriched brines.

Brine	4Ca	12Ca	37Ca	110Ca	4Mg	12Mg	37Mg	110Mg	4SO4	12SO4	37SO4	110SO4
$\text {Na}^+$	–	–	–	–	–	–	–	–	8	24	73	220
$\text {Ca}^{2+}$	4	12	37	110	–	–	–	–	–	–	–	–
$\text {Mg}^{2+}$	–	–	–		4	12	37	110	–	–	–	–
$\text {Cl}^-$	8	24	73	220	8	24	73	220	–	–	–	–
$\text {SO}_4^{2-}$	–	–	–	–	–	–	–	–	4	12	37	110
TDS [$\text {g/L}$]	0.5	1.4	4.14	12.2	0.4	1.2	3.5	10.5	0.6	1.7	5.2	15.7
Ionic strength	0.012	0.037	0.110	0.331	0.012	0.037	0.110	0.331	0.012	0.337	0.110	0.331

**Table 5:** Formation-water, Seawater, and Diluted-seawater impact on carbonate rock zeta potential and pH value.

Carbonate rock	Formation-water		Seawater		Diluted-seawater	
	Zeta-potential [mV]	pH	Zeta-potential [mV]	pH	Zeta-potential [mV]	pH
Indiana Limestone	9.3	7.49	− 6.6	8.92	− 16.8	9.87
Edward Limestone	11.2	7.58	− 3.1	8.93	− 9.2	9.90
Reservoir Carbonate	5.7	7.55	− 5.5	8.96	− 12.1	9.58
Austin Chalk	10.7	7.57	− 4.6	8.94	− 16.1	9.72
Silurian Dolomite	20.4	7.90	3.1	9.05	− 0.3	10.07

**Table 6:** Table of computed $\log K$ values for surface- and aqueous complexes in the carbonate surface complexation modeling.

Complex (secondary specie)		Basis species	logK @ 25$^\circ$C
>CaCO$_{3}^{-}$	$\rightleftharpoons$	>CaH$_2$O$^{+}$ + HCO$_3^-$ - H$^{+}$	7.10
>CaOH$^{0}$	$\rightleftharpoons$	>CaH$_2$O$^{+}$ + H$_2$O - H$^{+}$	12.90
>CaHCO$_3^{0}$	$\rightleftharpoons$	>CaH$_2$O$^{+}$ + H$_2$O	− 1.04
>CaSO$_4^{-}$	$\rightleftharpoons$	>CaH$_2$O$^{+}$ + SO$_{4}^{2-}$	− 2.90
>CO$_{3}$H$^{0}$	$\rightleftharpoons$	>CO$_3^{-}$ + H$^{+}$	− 4.90
>CO$_3$Ca$^{+}$	$\rightleftharpoons$	>CO$_3^{-}$ + Ca$^{2+}$	− 1.73
>CO$_3$Mg$^{+}$	$\rightleftharpoons$	>CO$_3^{-}$ + Mg$^{2+}$	− 1.24

## Data Availability

The datasets generated during and/or analyzed during the current study are available from the corresponding author upon reasonable request.
